# Importance of histopathological analysis and molecular genetics in a rare neonatal case of rhabdomyosarcoma

**DOI:** 10.1111/ajd.13849

**Published:** 2022-05-02

**Authors:** Prudence Gramp, Tania Zappala, Lena Von Schuckmann, Diane Payton, Laura Wheller

**Affiliations:** ^1^ Gold Coast University Hospital Southport Queensland Australia; ^2^ Queensland Children’s Hospital, University of Queensland South Brisbane Queensland Australia; ^3^ Queensland Children’s Hospital South Brisbane Queensland Australia; ^4^ Queensland Children’s Hospital, Royal Brisbane and Women’s Hospital Herston Queensland Australia

**Keywords:** child, dermatology, rhabdomyosarcoma

## Abstract

We present a case of a neonate who presented with multiple cutaneous and subcutaneous nodules, which was found to be metastatic embryonal rhabdomyosarcoma. Rhabdomyosarcoma is a soft tissue malignancy that usually occurs in children aged one to five but is rare in neonates. The histopathological analysis and molecular genetics are important in the classification of subtype and in guiding treatment options and informing prognosis.

## CASE

**FIGURE 1:**
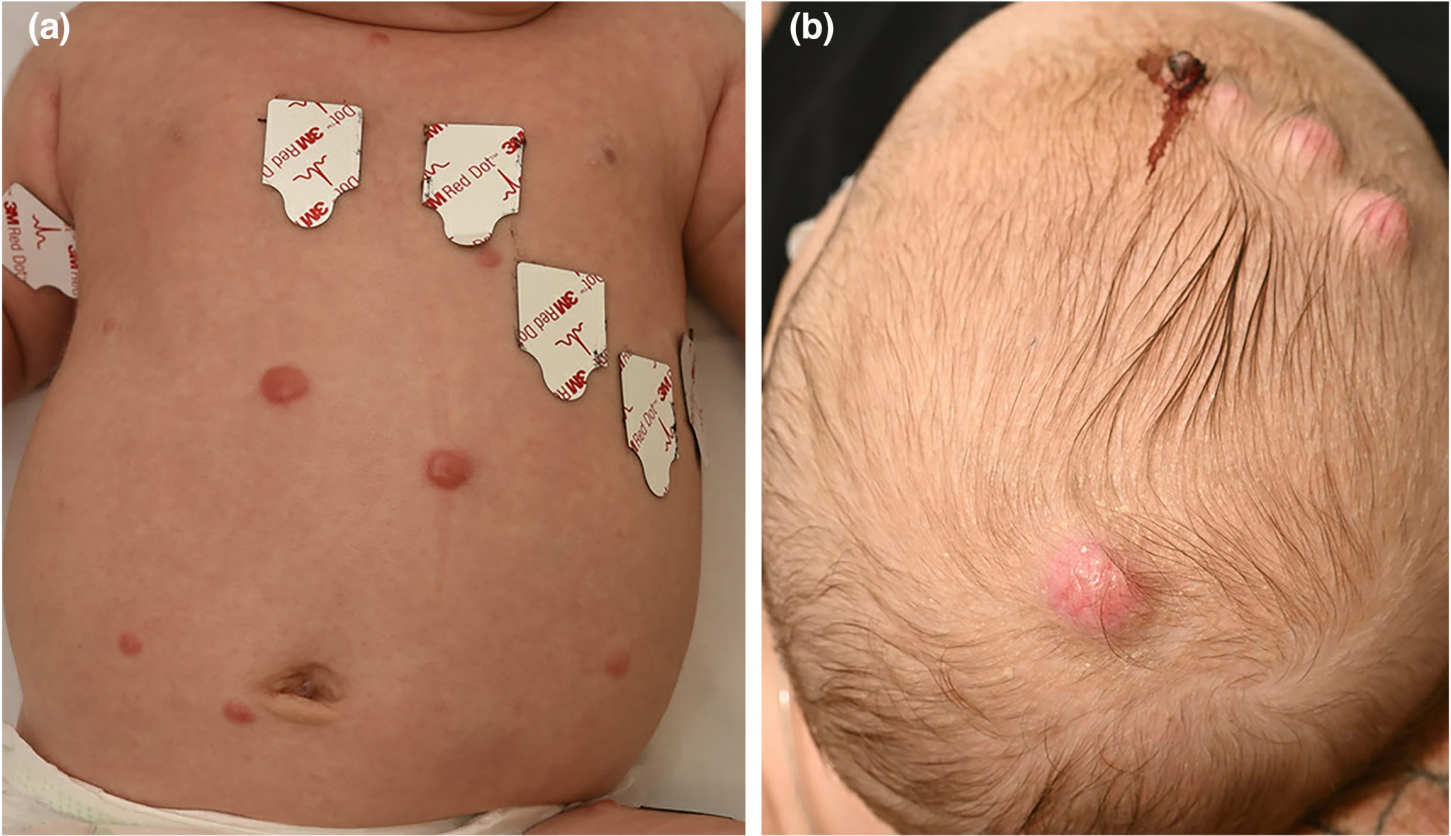
(a) Nodules on the abdomen. (b) Nodules on the scalp

A three‐week‐old infant presented with rapidly developing pink/erythematous to yellow/brown cutaneous and subcutaneous papules and nodules varying in size from 0.4 to several centimetres. The first lesion was noticed by his parents at 1 week of age. At the presentation to our dermatology clinic review at 6 weeks of age, the patient had numerous cutaneous and subcutaneous lesions (Figure 1a,b). The nodules were erythematous and firm and found on the limbs, scalp, torso, buttock and scrotum.

**FIGURE 2:**
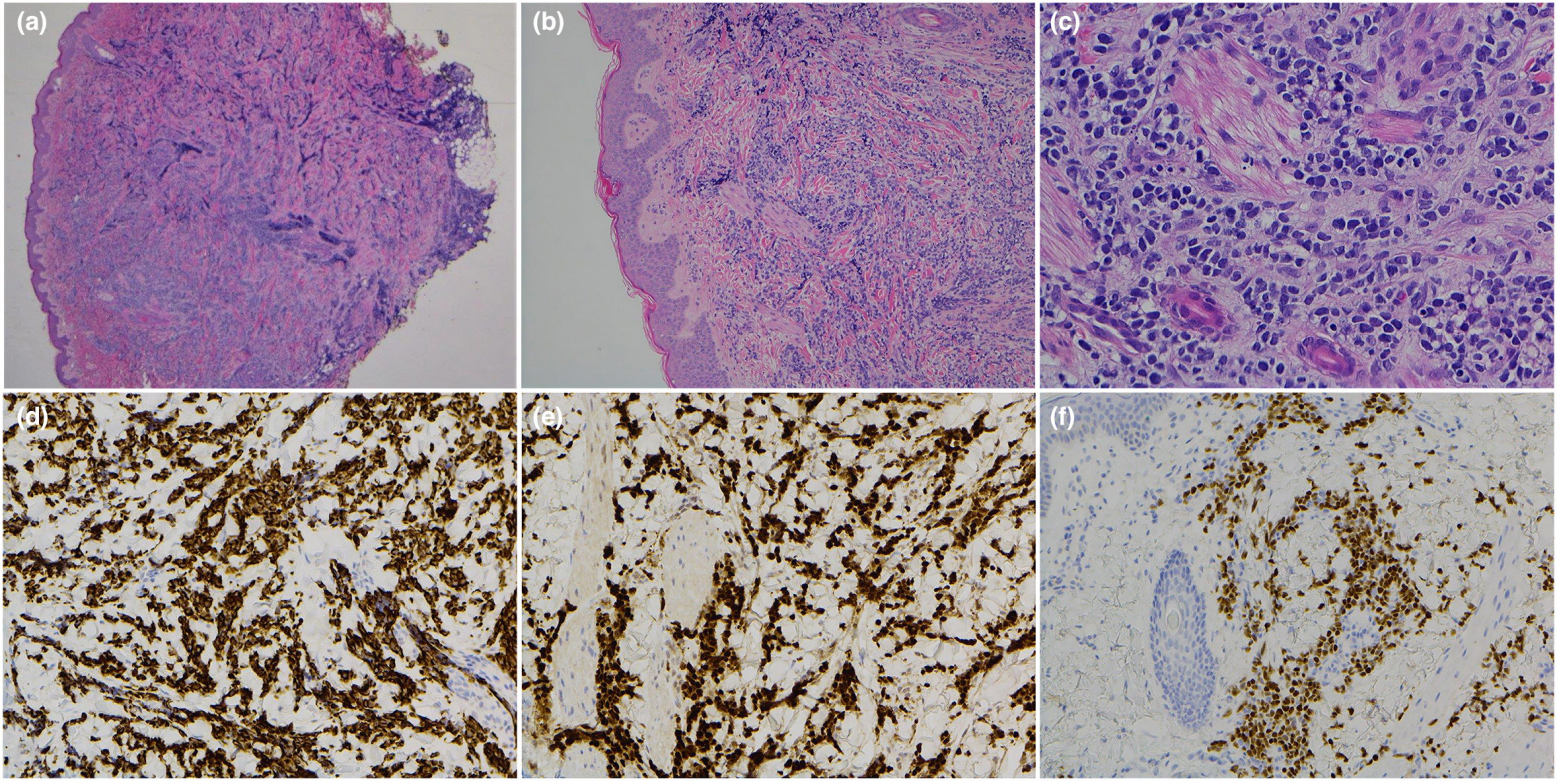
(a) (H&E x2): Full thickness dermal infiltrate of small tumour cells. (b) (H&E x10): Infiltrate between dermal collagen with sparing of adnexa. (c) (H&E x 40): Poorly differentiated tumour cells with hyperchromatic nuclei, ill‐defined minimal cytoplasm, mitoses and karyorrhexis. (d,e,f) (desmin, myogenin and MYOD1) positive in tumour cells

Initial investigation involved taking multiple biopsies of the back, debulking of an ear lesion and laryngoscopy to exclude airway nodules under general anaesthetic. Histopathology (Figure 2) showed an atypical cellular infiltrate as cords and nests with mild pleomorphism, ill‐defined cytoplasm and scattered mitotic figures involving the dermis and superficial subcutis. Multiple immunohistochemical stains were performed to differentiate the cell origin. MPO for granulocyte/neutrophil antibodies were negative. NSE for neuroendocrine lineage were negative. CD117 for mast cells and immature granulocyte lineage were negative. The positive immunoperoxidase for desmin, MYOD1 and myogenin (Figure 2) was consistent with rhabdomyosarcoma. Fluorescence in situ hybridisation (FISH) was performed on formalin‐fixed paraffin‐embedded tissue for FOXO1, which showed two fusions on chromosome 13 indicating no rearrangement of the FOXO1 gene. This is important in subtyping, where in alveolar subtypes the green and red signals seen in the FOXO1 gene would be broken apart.

**FIGURE 3:**
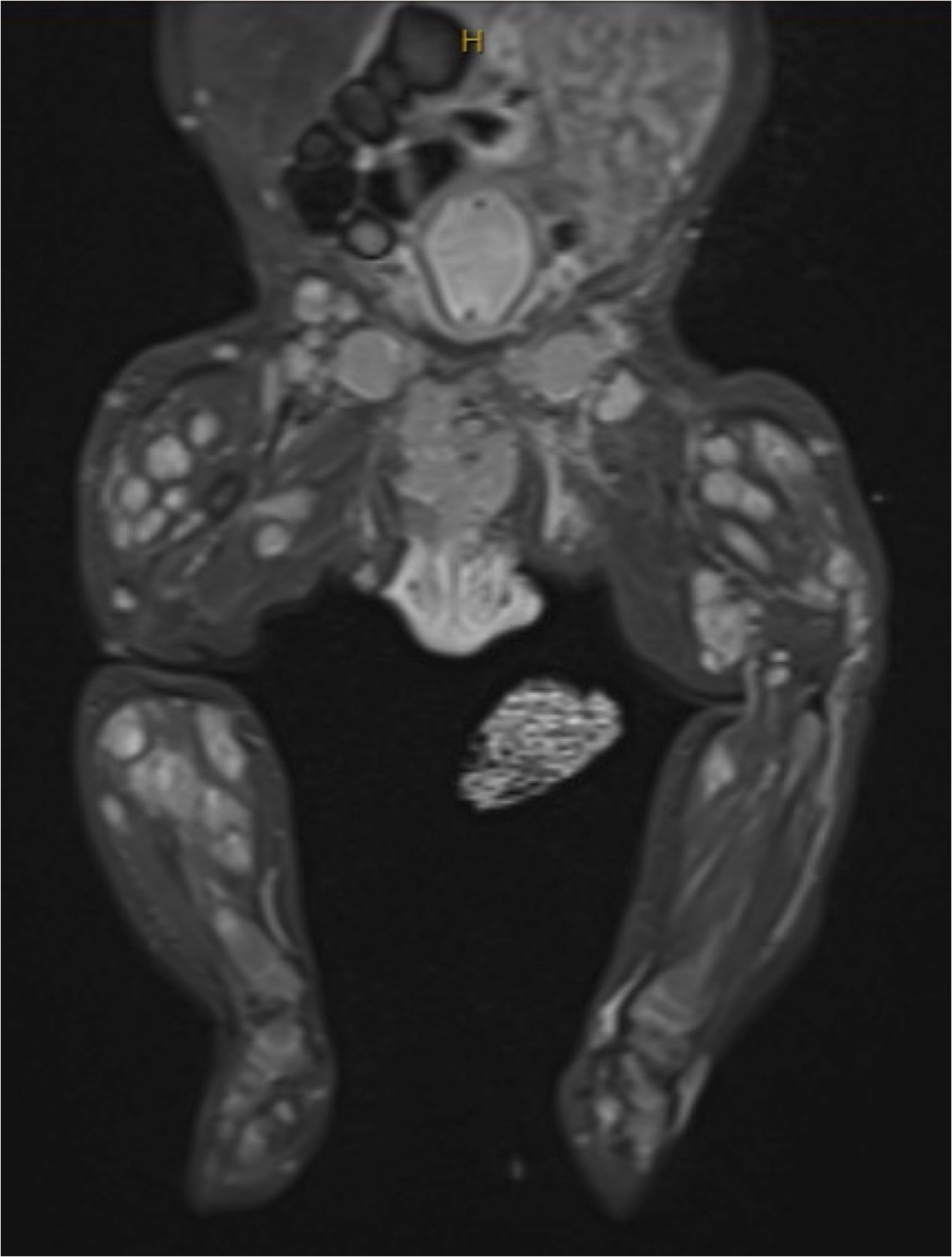
MRI imaging showing disseminated disease throughout the legs and abdomen

MRI brain, whole body and USS renal tract were performed, which showed extensive disseminated metastatic disease with innumerable muscle, cutaneous, subcutaneous tissue nodules and multiple osteolytic lesions most prominent in the right third metatarsal, but no central nervous system involvement (Figure 3). The diagnosis was confirmed as stage‐IV metastatic embryonal rhabdomyosarcoma with likely a urinary tract primary.

Rhabdomyosarcoma is rare with an incidence of 5 in 1 million in children and very rare in neonates.^1^, ^2^ It is a soft tissue tumour of primitive mesenchyme origin with incomplete myogenesis and while many arise in skeletal muscle, 25% of cases <15 years arise in the genitourinary tract.^1^, ^3^ Treatment involves chemotherapy, surgical removal and radiation.^4^, ^5^ There is less evidence for treatment in patients less than two years and infants <1 year old have poorer outcomes, thought to be due to higher rates of local control failure.^4^ The specific treatment regimen and prognosis is heavily reliant on histopathology and molecular genetics.^6^, ^7^, ^8^ There is poor prognosis with metastatic disease and infantile presentation; however, the lack of involvement of the central nervous system and negative FOXO1 gene rearrangement were favourable attributes in this case.^6^, ^7^, ^8^

There is variability in the classification of rhabdomyosarcoma with multiple credible classification systems and diagnostic methods. The minimum requirements for diagnosis include morphologic evaluation by H&E staining, and Myogenin, desmin and MYOD1 immunohistochemistry.^1^, ^8^ Table 1 shows a summary of important differentials that must be considered in similar cases and their relevant of the immunohistochemical stains useful for confirming diagnosis.^9^ The subtypes of rhabdomyosarcoma identified by a 2020 International Consensus paper are alveolar (higher‐risk subtype), embryonal (intermediate risk) and pleomorphic (lower risk).^8^

**TABLE 1:** Differential diagnoses for similar case presentations with relevant immunohistochemical findings

Differential diagnoses	Relevant negative or positive immunohistochemical findings
Congenital Myofibromatosis	Vimentin, alpha smooth muscle Actin positive
Extra‐skeletal Ewings	CD99 positive
Leukaemia/Lymphoma	LCA/CD45 positive
Malignant Peripheral Nerve Sheath Tumour	S100 positive
Metastatic Neuroblastoma	Chromogranin/CD56 positive
Rhabdoid Tumour	INI1 negative (i.e. no nuclear staining), myogenin/myoD1 negative
Rhabdomyosarcoma	INI1 Retained. Often high levels of myogenin and MYOD1. Desmin/muscle specific Actin positive.

The two main rhabdomyosarcoma subtypes are embryonal and alveolar, with embryonal being the most common in children, and especially in age groups <5 years.^7^ The alveolar subtype also has a poorer prognosis overall and has a strong expression of myogenin, whereas embryonal is variable (see Table 2).^7^ Also of note is the positive FOXO1 gene rearrangements in alveolar subtypes (see Table 2).^6^ This is of high prognostic value as there is only a 10% chance of survival with metastatic FOXO1 fusion positive rhabdomyosarcoma.^6^, ^9^ Pleomorphic and spindle cell subtypes are now recognised in the WHO classification of soft tissue tumours includes pleomorphic and spindle cell subtypes that are outlined in Table 2.^12^

**TABLE 2:** Comparative features of rhabdomyosarcoma subtypes

Rhabdomyosarcoma subtype	Embryonal rhabdomyosarcoma	Alveolar rhabdomyosarcoma	Pleomorphic rhabdomyosarcoma	Spindle cell rhabdomyosarcoma
Patient age	Usually <5 years unless spindle cell variant (more common in adolescents)	All ages, however, more common in >5 years old	Adults	Infantile, paediatric and adult
Site of origin	Head and neck, abdomen, genitourinary tract, scrotum	Extremities, trunk, head	Extremities	Head, neck, paratesticular and extremities
Genetic features	Loss of heterozygosity of short arm chromosome 11Heterogeneous expression on gene expression array	PAX3‐FOXO1 fusion PAX7‐FOXO1 fusion Homogenous expression on gene expression with fusion positive tumours	Complex genetic changes with copy number alterations and unbalanced structural alterations	NCOA2/VGLL2 gene fusions (infantile), MYOD1 gene mutations
Myogenin expression	Inconsistent reactivity: 0–80% of cells	Strong reactivity: 80–100% of cells	Focal positivity in ~50% of cases	Low level of myogenin expression
Prognosis	Favourable	Poor	Poor	Infantile – favourableMYOD1 mutant ‐ poor

While around 85% of alveolar RMS contain the FOXO1 rearrangement, a small number of alveolar RMS cases lack this rearrangement and so it must be considered that this case is a fusion negative metastatic alveolar RMS, given the strong myogenin/MYOD1 staining. There have been reported cases in the literature of congenital cutaneous RMS with both positive FOXO1 fusion, supporting a diagnosis of alveolar subtype, as well as without FOXO1 fusion supporting a diagnosis of embryonal RMS.^10^, ^11^

The patient received chemotherapy under oncology specialist supervision with vincristine, dactinomycin and cyclophosphamide (ARST1431 Regimen A ‐VAC only) and had treatment for multiple other complications including feeding support, otitis externa, urinary obstruction, hypertension, and a hypoxic brain injury as a complication of an infection. An MRI scan completed 6 weeks after the start of chemotherapy demonstrated dramatic reduction in the extent of metastatic disease, where the soft tissue lesions, including the subcutaneous tissue, intramuscular, pulmonary, pleural, penis, renal and nodal disease had resolved. Unfortunately, weeks after this last cycle of chemotherapy was completed the patient presented with signs of hydrocephalus and was found to have extensive leptomeningeal disease. Neurosurgery performed a VP shunt and referred to palliative care and sadly the patient died 1 month later.

## CONFLICT OF INTEREST

The authors declares that there is no conflict of interest.
